# The Transcription Factors *Six3* and *Six6* in Neuromedin‐S Neurons Differentially Affect Circadian Rhythms

**DOI:** 10.1002/jnr.70123

**Published:** 2026-04-05

**Authors:** Brooke M. Van Loh, Geneva A. Dunn, Lauren E. Chun, Meera M. Patel, Nay Chi P. Naing, Duong Nguyen, Alexandra M. Yaw, Michael R. Gorman, Jessica Cassin, Pamela L. Mellon, Hanne M. Hoffmann, Karen J. Tonsfeldt

**Affiliations:** ^1^ Department of Animal Science Michigan State University East Lansing Michigan USA; ^2^ Department of Obstetrics, Gynecology, and Reproductive Sciences, Center for Reproductive Science and Medicine University of California San Diego La Jolla California USA; ^3^ Department of Psychology University of California San Diego La Jolla California USA; ^4^ Center for Circadian Biology University of California San Diego La Jolla California USA

**Keywords:** circadian rhythms, neuromedin‐s, reproduction, Six3, Six6, suprachiasmatic nucleus

## Abstract

Circadian rhythms are ~24‐h rhythms generated by the suprachiasmatic nucleus (SCN) in the mammalian hypothalamus. The regulation of circadian rhythms and downstream processes is highly dependent on the proper development and function of the SCN. *Six3* and *Six6* are homologous homeodomain transcription factors that have been shown to be required for SCN development; intriguingly, both *Six3* and *Six6* remain expressed in the adult SCN. To determine the role of *Six3* and *Six6* in the SCN after neurogenesis, we used Cre‐lox to conditionally knockdown either *Six3* or *Six6* from cells that express neuromedin‐S (NMS), a neuropeptide expressed in approximately half of SCN neurons. We found that the *Nms* ^*cre*^ allele turns on in the SCN after embryonic Day 16.5, limiting Cre‐lox‐mediated loss of *Six3* or *Six6* to the period after SCN neurogenesis. Using this approach, we hypothesized that *Six3* and *Six6* in NMS neurons regulate SCN circadian output and resulting reproductive function in males and females. Loss of *Six6* from NMS neurons had no impact on puberty and reproduction. While loss of *Six3* from NMS neurons had no effect in females, we found significantly decreased sperm motility in males, potentially through direct effects of *Six3* in the testis. Loss of *Six3,* but not *Six6*, in NMS neurons resulted in shortened wheel‐running periods in constant darkness, indicating a shortening of the endogenous rhythm within the SCN. Together, these data indicate a role of *Six3* in determining the circadian period, suggesting differing functions of *Six3* and *Six6* in the adult SCN.

## Significance Statement

Homeobox genes *Six3* and *Six6* are highly homologous transcription factors critical for the development of the forebrain. Expression of *Six3* and *Six6* is maintained in the adult suprachiasmatic nucleus, the central circadian pacemaker. We demonstrate that *Six3* and *Six6* have distinct roles in the SCN, using conditional knockout of *Six3* or *Six6* in neuromedin‐S‐expressing cells, which include approximately half of the SCN. These findings advance our understanding of circadian and neuroendocrine regulation by *Six3* and *Six6,* providing new insight into congenital hypothalamic dysfunction.

## Graphical abstract

*Six3* and *Six6* are necessary for the development of the suprachiasmatic nucleus (SCN), and both remain expressed after SCN neurogenesis. We implemented a Neuromedin‐S (NMS)‐cre mouse model to conditionally knockout either *Six3* or *Six6* from the SCN to determine the post‐proliferative roles of these transcription factors in hypothalamic and circadian function.

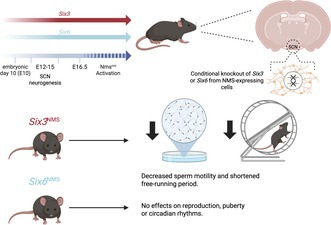

## 1 Introduction

Circadian rhythms are approximately 24‐h rhythms that coordinate biological processes throughout the body. In mammals, these circadian rhythms are synchronized by the suprachiasmatic nucleus (SCN) in the hypothalamus. There, a molecular clock composed of a transcription‐translation feedback loop of clock proteins including BMAL1, CLOCK, Periods (PER1, PER2, PER3), and Cryptochromes (CRY1, CRY2) generates cell‐autonomous circadian rhythms. When components of the molecular clock or SCN function are disrupted, downstream rhythms including reproduction (Alvarez et al. 2008; Miller et al. 2004; Miller and Takahashi 2014; Tonsfeldt et al. 2021; Williams Iii and Kriegsfeld 2012), sleep (Mistlberger 2005; Stephan and Nunez 1977), and metabolism (Froy 2010; Gangwisch 2009; Marcheva et al. 2009) are impaired. The neurogenesis and formation of the SCN rely on homeodomain transcription factors, including homeoboxes *Sine oculis* 3 (*Six3*) and *Sine oculis* 6 (*Six6*) (Conte et al. 2005; Pandolfi, Breuer, et al. 2019; VanDunk et al. 2011). Interestingly, the SCN retains expression of *Six3* and *Six6* in adulthood; however, any post‐developmental function of these genes is unknown (Conte et al. 2005; Hatori et al. 2014; VanDunk et al. 2011; Wen et al. 2020).

*Six3* and *Six6* are highly homologous, and disruption of either during the developmental period negatively impacts SCN formation, circadian regulation, and reproduction. In mice, homozygous loss of *Six3* results in failure to develop the anterior portion of the head and brain, resulting in fetal death (Lagutin et al. 2003). Previous work has shown that flox‐mediated recombination of *Six3* around embryonic Day (E) 12.5 (Zhu et al. 2001) using a neural‐specific driver, *Synapsin* ^*cre*^, results in irregular circadian rhythms in both male and female mice (Hoffmann et al. 2021; Meadows et al. 2022). Homozygous loss of *Six6* leads to variable development of the optic nerves, optic chiasm, and SCN, resulting in impaired or absent behavioral rhythms (Clark et al. 2013).

*Six3* and *Six6* are also regulators of the hypothalamic–pituitary‐gonadal axis. Briefly, gonadotropin‐releasing hormone (GnRH) neurons in the hypothalamus stimulate the release of luteinizing hormone (LH) and follicle‐stimulating hormone (FSH) from the pituitary that together control reproduction. *Six3* heterozygous females are profoundly subfertile and very few are able to produce litters, likely due to a 40% reduction in GnRH neurons and pituitary dysmorphology (Bando et al. 2022; Pandolfi et al. 2018). *Six3* ^*fl/fl*^ *‐Synapsin* ^*cre*^ female mice also display a reduction in fertility, disrupted estrous cyclicity, and impairment of the preovulatory LH surge, a process mediated by the SCN (Hoffmann et al. 2021). Homozygous loss of *Six6* results in infertility and a 90% reduction in GnRH neurons (Larder et al. 2011). In most of these rodent models, it is impossible to differentiate the developmental actions of *Six3* and *Six6* from distinct roles in the adult animal.

The continued expression of *Six3* has been investigated in the *Six3* ^*fl/fl*^ *‐Synapsin* ^*cre*^ mouse, where there were notable alterations of circadian behavior and subfertility in females (Hoffmann et al. 2021). However, as *Synapsin* ^*cre*^ is expressed in most mature neurons as well as extra‐central nervous system tissues, it is unclear which of these effects are due to the loss of *Six3* in the SCN specifically. To our knowledge, there has been no investigation into the post‐developmental role of *Six6*. We hypothesized that, in addition to the known developmental role of *Six3* and *Six6*, these two transcription factors are also involved in post‐developmental regulation of circadian function and downstream circadian processes. To test this hypothesis, we utilized a conditional knockout model wherein Cre recombinase was expressed under the control of a Neuromedin‐S (NMS) promoter, a neural peptide widely and predominately expressed in the SCN in the late‐infantile and early juvenile stages (Lee et al. 2015; Mori et al. 2008; Van Loh et al. 2023). We created conditional knockouts using floxed *Six3* or *Six6* mice, which allowed us to investigate the role of *Six3* and *Six6* in the SCN after neurogenesis.

## 2 Materials and Methods

### 2.1 Mice

All animal procedures were performed according to protocols approved by the University of California, San Diego Institutional Animal Care and Use Committee and the Institutional Animal Care and Use Committee of Michigan State University. Mice were maintained on a light/dark cycle of 12 h light, 12 h dark with light parameters as described previously (Van Loh et al. 2023). *Six6* ^*flox/flox*^ mice (RRID:MMRRC_068240‐UCD; Pandolfi, Tonsfeldt, et al. 2019) were crossed with heterozygous *Nms* ^*cre*^ mice (RRID:IMSR_JAX:027205; Lee et al. 2015) to generate mice discussed here as *Six6* ^*NMS*^. Similarly, *Six3* ^*flox/flox*^ (RRID MGI:6507739; Lagutin et al. 2003) mice were crossed with *Nms* ^*cre*^ mice to generate *Six3* ^*NMS*^ mice; or *Vip* ^*cre*^ mice (RRID:IMSR_JAX:010908, sourced from Jackson Labs) to generate *Six3* ^*VIP*^. In all conditional knockouts, Cre was maintained as heterozygous to avoid off‐target effects of the Cre allele (Joye et al. 2020). Cre negative, flox/flox littermates were used for controls (denoted *Six3* ^*fl/fl*^ and *Six6* ^*fl/fl*^). The Ai14Rosa tdTomato reporter mice (RRID:IMSR_JAX:007914, sourced from Jackson Labs) were crossed with *Nms* ^*cre*^ mice to create *tdTomato* ^*+/wt*^ *Nms* ^*cre*^ (*tdTomato* ^*NMS*^ mice) to visualize Cre‐containing neurons. Period2::Luciferase (PER2::LUC) mice were purchased from JAX (RRID:IMSR_JAX:006852) and crossed with *Six6* ^*NMS*^ mice for at least two generations. The mice used for the resulting studies were *Nms* ^*cre*^ *Six6* ^*fl/fl*^ PER2::LUC^+/−^; control mice for these studies were *Six6* ^*fl/fl*^ PER2::LUC^+/−^. Genotyping primer sequences are in Table 1. Mice were screened for germline recombination using the primer combinations listed, and germline recombined animals were excluded from the studies.

**TABLE 1:** Genotyping primers.

Mouse	Primers
NMS‐Cre	FWD: CCA AGT TAG CCT TCC ATA CAC C
	REV: AGA CGG CAA TAT GGT GGA AAA T
VIP‐Cre	FWD: GCA TTA CCG GTC GTA GCA ACG AGT G
	REV: GAA CGC TAG AGC CTG TTT TGC ACG TTC
Six3 Flox	FWD: TGC CCC CTG CTA AAG AGC CAG T
	REV1: TAG GGA CAG GCA CGG AGG GTT G
	REV2: ATG CCC ACA TTG TCG GCC CAT G
Six6 Flox	FWD: GAA GCC CTT AAC AAG AAT GAG TCG G
	REV1: TCC CTT TGA ATT TGG GTC CCT G
	REV2: CTT CGG AAT AGG AAC TTC GGT
Rosa tdTomato	FWD1: GGC ATT AAA GCA GCG TAT CC
	REV1: CTG TTC CTG TAC GGC ATG G
	FWD2: CCG AAA ATC TGT GGG AAG TC
	REV2: AAG GGA GCT GCA GTG GAG TA

### 2.2 Single‐Cell RNA (scRNA) Analysis

Single‐cell RNA sequencing data were gathered using HypoMap (Steuernagel et al. 2022), a publicly available dataset using 17 published 10X Genomics and Drop‐seq mouse hypothalamus datasets. We utilized the CELLxGENE interface to probe the data. We sub‐selected SCN neurons following (Correa‐da‐Silva et al. 2025), first by selecting for neuronal cells, and then selecting for the SCN region. We then queried the set of 21,985 SCN neurons for expression of *Nms*, *Six3*, and *Six6*. Cells were considered positive if they had more than zero counts of the transcript.

### 2.3 tdTomato Reporter Analysis

*Nms* ^*cre*^ male mice were mated with Rosa‐tdTomato^+/+^ females and checked daily for vaginal plugs. Pups were retrieved on E16.5 and the heads drop‐fixed in 4% PFA overnight at 4°C. Tails were genotyped for *Cre* and *Sry*. After fixation, heads were sunk in 30% sucrose, embedded in O.C.T. (Tissue‐Tek, Sakura) and stored at −80°C until sectioning. Heads (*n* = 3, 2 female, 1 male) were sectioned coronally at 20 μm in two series. One series underwent gentle washing in PBS for 20 min followed by incubation in 1 μg/mL DAPI for 6 min, followed by another 20‐min PBS wash prior to coverslipping. Slides were imaged using a Zeiss Axio Imager M2. To show expression in adulthood, the brain from one 8‐week old mouse was drop‐fixed in 4% PFA as above. The brain was sectioned at 40 μm, and sections containing the SCN were mounted on slides. The SCN was visualized on an Andor BC43 (Oxford Instruments).

### 2.4 Cell Culture and Transient Transfections

A detailed protocol on cell culture and transient transfections has been previously described (Meadows et al. 2022). In brief, mouse fibroblast NIH3T3 (RRID:CVCL_0594) cells were incubated in 10 cm plates in an incubator at 37°C with 5% CO_2_ and maintained in complete media containing Dulbecco's Modified Eagles Media (DMEM), 10% fetal bovine serum (FBS), and 1% penicillin–streptomycin. Cells were transfected with a *Six6* expression vector (Larder et al. 2011) or empty vector (pcDNA) and a reporter vector: Mouse *Bmal1*‐luc (a gift from Steven Brown [Addgene plasmid # 46824; RRID:Addgene_46824; Brown et al. 2005]) or Pgl empty vector; mouse *Per2*‐luc (Period 2; a gift from Joseph Takahashi [Addgene plasmid # 48746; http://n2t.net/addgene:48746; RRID:Addgene_48746; Yoo et al. 2005; Albrecht et al. 1997]) or Pgl2 empty vector; rat *Avp*‐luc (arginine vasopressin; a gift from Robert Shapiro 2000) or Pgl2 empty vector; or mouse *Vip*‐luc (vasoactive intestinal peptide; Hatori et al. 2014) or Pgl3 empty vector. To control for transfection efficiency, a reporter plasmid containing β‐galactosidase constitutively driven by the Herpes virus thymidine kinase promoter (TK‐βgal) was used.

On Day 1, cells were plated at 30,000 cells/well in a 12‐well plate and incubated overnight to allow cell adherence. On Day 2, cells were transfected with the following amounts of plasmid: TK‐βgal at 20 ng/well, luciferase plasmids or empty luciferase backbone at 400 ng/well, and pcDNA‐empty vector (EV) or *Six6* expression vectors at 400 ng/well. Transfections were performed using the reagent Polyjet per manufacturer's instructions (SignaGen Laboratories). On Day 3, 24‐h post‐transfection the media was changed to fresh, complete media. On Day 4, 24 h post media change, the cells were harvested in lysis buffer (100 mM potassium phosphate pH 7.8 and 0.2% Triton X‐100) and plated in 96‐well plates and read for luciferase and β‐gal assays in a Luminometer microplate reader (Glomax, Promega). Within each well, luciferase values were normalized to TK‐βgal values, then triplicate luciferase/TK‐βgal values were averaged. Luciferase was normalized to the luciferase backbone for both *Six6* and pcDNA. Fold change is displayed as normalized Six6/pcDNA.

### 2.5 Pubertal Onset

Beginning at postnatal Day 21, pubertal onset was established by visual inspection of preputial separation (PPS) in males and vaginal opening (VO) in females, as described previously (Hoffmann 2018). Examination occurred between zeitgeber time (ZT) 2–4, and the assessor was blind to genotype. Body weight was recorded on the day pubertal onset was observed.

### 2.6 Female Fertility Assessments

Estrous cyclicity was monitored by vaginal lavage with 20 μL H_2_O or saline daily between ZT 3–5 for 15–18 consecutive days in 12–14‐week‐old mice. The lavage solution was dried on a glass slide and stained with 0.1%–0.5% methylene blue. Cytology was examined and scored by two independent, blinded observers. Smears were scored for the presence of leukocytes (diestrus), nucleated epithelial (proestrus), and cornified epithelial cells (estrus) (Byers et al. 2012). To assess fecundity, virgin 15–17‐week‐old female *Six3* ^*NMS*^ or *Six6* ^*NMS*^ mice were housed with 10–16‐week‐old male mice for 76 days. Mating cages were checked daily in the morning. Days to first litter, how many litters occurred in 76 days, and the number of pups per litter at birth were recorded.

### 2.7 Induced Luteinizing Hormone (LH) Surge

Female mice underwent bilateral ovariectomy. Bosch et al. (2013), 4 days later mice received a subcutaneous dose of 0.25 μg estradiol (E2; Cat No. E8875, Sigma Aldrich) in sesame oil (Cat. No. S3547, Sigma Aldrich) between ZT 3–4. The following day, mice received a subcutaneous dose of 1.5 μg estradiol in sesame oil between ZT 3–4. The following evening (~30 h after injection), mice were sacrificed at lights out at ZT 12. Whole blood was collected, allowed to clot for 90 min, and spun for 20 min at 20,000 × g to separate serum. Serum was stored at −80°C until analysis. All procedures were performed blind to genotype.

### 2.8 LH Assay

LH was measured using the ultra‐sensitive LH assay (Kreisman et al. 2022). Briefly, serum samples were diluted 1:17 in assay buffer (PBS + 0.2% BSA). The sandwich ELISA was performed using LH 518B7 (UC Davis, Cat. no. 518B7, lot 13; RRID:AB_2665514) as a capture antibody at 1.0 μg/mL and biotinylated 5303 SPRN‐5 (Medix Biochemica RRID:AB_2784503) as a detection antibody at 4.379 μg/mL. A standard curve from 1 to 0.0019531 ng/mL was generated using Golden West Biosolutions, catalog No. TLIA1053.03. The plate was analyzed using a Tecan infinite 200 Pro plate reader at 490 nm minus the 650 nm background. A four‐parameter logistic curve was generated using MyAssays.com website (http://www.myassays.com/). The intra‐assay CV was 7.22%, and the inter‐assay CV was 6.21%.

### 2.9 Male Sperm Count and Plugging Assay

Testes, seminal vesicles, and epididymis were collected and weighed. Sperm was collected from the epididymis of male mice in 37°C M2 media (Sigma). The epididymis was cut in half and sperm were expelled by gently pressing down on the epididymis and then left in M2 media at room temperature for 15 min. The number of motile sperm was counted from a 10 μL aliquot of the M2 media containing sperm by using a hemocytometer. The hemocytometer was then placed on a heat block at 55°C for 5 min to immobilize all sperm. The total number of intact and immobile sperm was counted to determine percent motility. The second epididymis was cut into small pieces and left for 15 min at room temperature in M2 media. The solution was gently homogenized often to help liberate the sperm. The solution was filtered using a cell streamer (70 μm, Falcon), and sperm were diluted 1:10 with MQ before counting the total number of sperm heads.

To assess male plugging behavior, *Six3* ^fl/fl^ or *Six3* ^*NMS*^ male mice were paired with a virgin WT female for 10 days. During the assay period, the females and bedding were checked daily between ZT 2 and 3.

### 2.10 Corticosterone Sampling and Assay

Pair‐housed mice were allowed to acclimate in chambers on a 12:12 light:dark cycle for 2 weeks. Tail bleeds were collected every 4 h for 24 h into capillary tubes, and serum was collected and stored at −20 C until assay. Approximately 10 μL of tail blood was collected using a capillary (Drummond Scientific) from each mouse at each timepoint. To minimize the number of times that the tail was cut for repeated blood collection, cuts from previous timepoints were re‐opened with a cotton swab soaked in saline. After the 24‐h collection in LD, mice were transitioned to constant darkness (DD) for 4 weeks before collection in DD. Because the activity rhythms of the mice were not monitored, tail bleeds in DD were collected based on clock time rather than the time relative to the activity onset of each animal. Corticosterone was measured from serum using the DetectX Corticosterone ELISA kit (Arbor Assays) according to the manufacturer's instructions. For all samples, 4 μL of serum was diluted 1:400 in dissociation reagent prior to loading on the assay. In some cases, time points from some individual animals were lost due to unanticipated collection issues. The plates were visualized using an iMark microplate reader (Bio‐Rad) and final concentrations were quantified using a four‐parameter logistic curve on MyAssays.com.

### 2.11 Wheel‐Running Behavior

Female and male mice aged 8–12 weeks were singly housed in cages containing metal running wheels and wheel revolutions were monitored using magnetic sensors as previously described (Van Loh et al. 2023). Genotypes were randomly distributed in cages in a light‐tight cabinet with programmable lighting conditions. Rooms were monitored for temperature and humidity. Food and water were available *ad libitum* during the entire experiment. After 1‐week acclimation to the polypropylene cages (17.8 × 25.4 × 15.2 cm or 33.2 × 15 × 13.2 cm) containing a metal running wheel (11.4 cm diameter or 11 cm diameter, respectively), locomotor activity rhythms were monitored with a VitalView data collection system (Version 4.2, Minimitter) that integrated in 6 min bins the number of magnetic switch closures triggered by half wheel rotations or full wheel rotations, respectively. Running wheel activity was initially monitored for 2 weeks in a standard 12 h light/12 h dark cycle (LD), whereafter the mice were monitored for 4 weeks in constant darkness (DD), with wheel running data analyzed from Weeks 2–4 (14 days) in DD. In some cases, mice were exposed to a 2 week “skeleton” light paradigm (1 h light: 11 h dark: 1 h light: 11 h dark) in between LD and DD. We found no evidence of masking in the skeleton photoperiod phase angle between *Six3* ^*fl/fl*^ and *Six3* ^*NMS*^ (−0.05 ± 0.50 vs. −0.04 ± 0.38 h, *t*(11) = −0.036, *p* = 0.972, *n* = 6–7) or *Six6* ^*fl/fl*^ and *Six6* ^*NMS*^ (0.37 ± 0.13 vs. 0.23 ± 0.20 h, *t*(16) = 1.688, *p* = 0.114, *n* = 8) so we excluded skeleton photoperiods from future experiments. Cage changes were scheduled for 2–4‐week intervals. Light intensity varied between 268 and 369 l× inside the mouse cage with the wheel. Wheel running activity was analyzed using ClockLab Analysis (ActiMetrics) by an experimenter blind to experimental group. Circadian period (tau, or free‐running rhythm) was analyzed by constructing a least‐squares regression line through a minimum of 14 daily activity onsets in constant darkness. Rhythm strength was assessed using the amplitude of a *χ* ^2^ periodogram from the first 2 weeks of LD or the final 2‐week period of DD. Alpha was determined based on the length of time that activity was greater than 20% of the peak activity. Onset precision was calculated as the standard deviation of onsets compared to the time of lights on (LD) or the predicted tau (DD). Mice were compared to their littermate controls, and male and female mice were grouped for analysis except where noted.

### 2.12 Ex Vivo Tissue Recordings of PER2::LUC Expression

For circadian rhythm organotypic explant studies, tissues from mice expressing the PER2::LUC circadian reporter were collected and analyzed as previously described (Yaw et al. 2020). Proestrus Six6^NMS^ PER2::LUC and PER2::LUC control females were placed on LD and euthanized at ZT 3–4. The brain, pituitary, ovaries, and uterus were removed immediately and placed in an ice‐cold, CO_2_ saturated Hank's Balanced Salt Solution (HBSS) for approximately 1 h. Using a Vibratome (Leica), tissue sections of 300 μm were collected and the indicated brain region was dissected from the slices in ~2 × 2 mm squares and placed on a 30 mm Millicell membrane (Millipore‐Sigma) in a 35 mm cell culture plate containing 1 mL Neurobasal‐A Medium (Gibco) with 1% Glutamax (Gibco), B27 supplement (2%; 12349‐015, Gibco), and 1 mM luciferin (BD Biosciences). The lid was sealed to the plate using vacuum grease to ensure an air‐tight seal. Plated tissues were loaded into a LumiCycle luminometer (Actimetrics) inside a 35°C non‐humidified incubator at ZT 6–6.5, and recordings were started. The bioluminescence was counted for 70 s every 10 min for 6 days (Day 1–Day 7 of recording time). PER2::LUC rhythm data were analyzed using LumiCycle Analysis software (Actimetrics) by an experimenter blind to experimental group. Data were detrended by subtraction of the 24‐h running average, smoothed with a 2‐h running average, and fitted to a damped sine wave (LM Fit, damped). Period was defined as the time in hours between the peaks of the fitted curve.

### 2.13 RNAscope (Multiplex In Situ Hybridization Assay)

Animals were sacrificed between ZT 8–11. Brains were flash frozen, sectioned at 20 μm, and stored at −80°C. Slides containing the SCN underwent multiplex in situ hybridization detection of mouse ( *Mus musculus* ) mRNAs with RNAscope LS Multiplex Fluorescent Reagent Kit (Advanced Cell Diagnostics) for 3‐plex assay in addition to RNAscope LS 4‐Plex Ancillary Kit (Advanced Cell Diagnostics) for 4‐plex assay following the vendor's standard protocol for FFPE tissue sections with minor modifications. RNAscope assays were performed on a Leica Bond autostainer as previously described (Sempere et al. 2020; Van Loh et al. 2023) with the following probes: RNAscope 2.5 LS Probe—Mm‐Avp‐C2 (arginine vasopressin [Avp] mRNA, cat no. 401398‐C2); RNAscope 2.5 LS Probe—Mm‐Vip‐C3 (vasoactive intestinal polypeptide [Vip] mRNA, cat no. 415968‐C3); and RNAscope 2.5 LS Probe—Mm‐Nms‐C4 (neuromedin S [Nms] transcript variant 1, cat no. 472338‐C4). Stock Mm‐Nms‐C4 probe was diluted at 1:50 in a pre‐diluted C1 probe as recommended by the vendor, whereas stock Mm‐Avp‐C2 and Mm‐Vip‐C3 were further diluted to 1:100 in appropriate pre‐diluted C1 probe due to saturating signal in the pilot experiment and tissue slides were counterstained with DAPI. Sections were imaged using a Zeiss Apotome.2 equipped with a Zeiss AxioCam 506 (Zeiss, Germany). Qupath (Bankhead et al. 2017) was used to automate cell detection based on DAPI staining. Cells were classified based on detection of subcellular objects indicating *Avp*, *Vip*, and *Nms* mRNA. Results were obtained per single SCN hemisphere and averaged by animal (2–4 hemispheres per animal).

### 2.14 Statistical Analysis

Statistical analyses were performed with GraphPad Prism 10. Sample sizes were determined based on previous studies in our laboratories or pilot studies. Outliers were identified using ROUT analysis (*Q* = 1%). Data were analyzed using unpaired *t*‐test, Mann–Whitney *U*‐test, one‐way ANOVA or two‐way ANOVA, followed by post hoc analysis where appropriate. *p < 0.05, **p < 0.01, ***p < 0.001, and ****p < 0.0001.

## 3 Results

### 3.1 SCN *Nms‐*Expressing Neurons Also Express *Six3* and *Six6*

**FIGURE 1:**
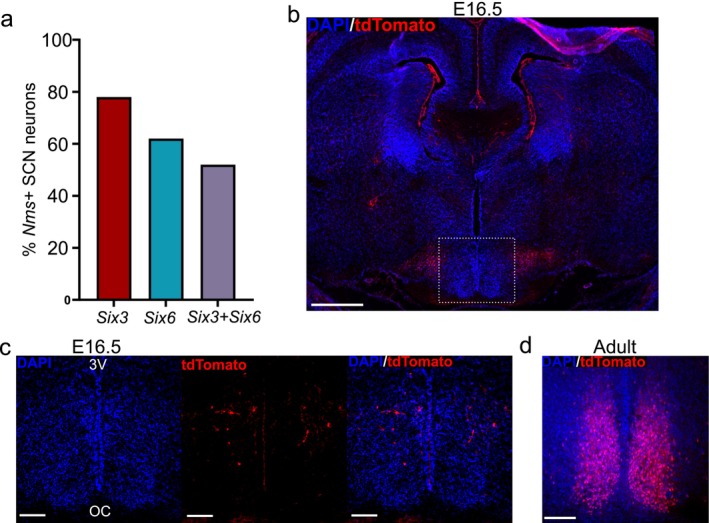
*Nms*, *Six3*, and *Six6* expression in the SCN. (a) Percentage of SCN *Nms +* neurons that contain *Six3*, *Six6*, and both *Six3* and *Six6*, using published scRNASeq data from HypoMap (Steuernagel et al. 2022). Data are expressed as a percentage of the total number of *Nms*‐expressing SCN neurons (6123 neurons). (b) Expression of *Nms* ^*cre*^ (as indicated by a tdTomato reporter) at E16.5. tdTomato representing *Nms* ^*cre*^‐mediated recombination is seen highly expressed far medial to the 3rd ventricle (3 V) Scale bar = 500 μm. (c) Representative closer magnification of the SCN, of outlined area in (b). (d) Expression of *Nms* ^*cre*^ (as indicated by a tdTomato reporter) at 8 weeks of age. Images are representative of *n* = 3. Scale bar = 100 μm. 3V, third ventricle; OC, optic chiasm.

SIX3 and SIX6 are highly expressed in the adult SCN (Hatori et al. 2014; VanDunk et al. 2011), but the overlap with each other and NMS is unknown. Using published single‐cell RNA sequencing data from HypoMap containing 17 individual scRNA datasets of mouse male and female hypothalamus (Steuernagel et al. 2022), we examined the overlap between *Nms, Six3*, and *Six6* expression in 21,985 SCN neurons. We found that 28% of SCN neurons express *Nms*, consistent with previous reports (Lee et al. 2015). Of *Nms +* neurons, 78% express *Six3*, 62% express *Six6*, and 52% express both *Six3* and *Six6* (Figure 1a). The cells that contain *Nms, Six3, and Six6* represent approximately 14% of SCN neurons. To compare our results with the previous findings in the *Six3* ^*fl/fl*^ *‐Synapsin* ^*cre*^ mouse, we also evaluated *Syn1* expression in these neurons and found that a larger population of SCN neurons express *Syn1* (37%), that 56% of *Nms +* neurons express *Syn+*, and 67% of *Syn1+* neurons express *Nms*. These results indicate that the *Nms* ^*cre*^ targets a distinct population of the SCN compared to *Synapsin* ^*cre*^. Having established that approximately half of *Nms* neurons express *Six3* and *Six6*, we then wanted to determine the developmental timing of *Nms* ^*cre*^ expression in the SCN. We used the *Nms* ^*cre*^ mice crossed with a Cre‐dependent tdTomato reporter to visualize neurons expressing Cre, as the tdTomato signal is expressed following *Nms* ^*cre*^‐mediated recombinase activity. To determine if *Nms* ^*cre*^ turns on after SCN neurogenesis, we examined the SCN of E16.5 *tdTomato* ^*NMS*^ embryos (Figure 1b,c). We observed no tdTomato‐containing cells in the SCN in either males or females (*n* = 3, 2 females), in contrast to robust tdTomato expression in the adult SCN (Figure 1d). We did observe tdTomato‐containing populations lateral to the SCN, indicating that there are areas of *Nms* ^*cre*^ expression at E16.5. We concluded that the activation of *Nms* ^*cre*^, and presumably NMS expression, occurs subsequent to SCN neurogenesis (~E12‐E15) (Kabrita and Davis 2008; Shimada and Nakamura 1973; VanDunk et al. 2011). Therefore, targeting *Six3* and *Six6* using *Nms* ^*cre*^ would allow us to isolate only those effects which occur after SCN neuron cell proliferation is complete and which are unlikely to influence the development of the SCN itself.

### 3.2 SIX6 Regulates Circadian Clock Genes *Per2* and *Bmal1,* and Neurotransmitter *Vip*

**FIGURE 2:**
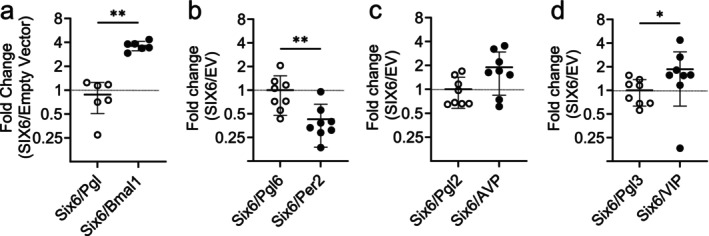
SIX6 acts on the promoters of SCN transcripts in vitro. Transient transfections of NIH3T3 cells with (a) *Bmal1*‐luciferase (*n* = 6), (b) *Per2*‐luciferase (*n* = 8), (c) *Avp*‐luciferase (*n* = 8) and (d) *Vip*‐luciferase (*n* = 8) or empty luc vector with *Six6* overexpression. Data were normalized to the empty SIX6 expression vector. Data were analyzed using Mann–Whitney U Test. Data expressed as mean ± SD. Significance indicated by *p < 0.05, **p < 0.01.

SIX3 can regulate molecular clock and SCN peptides in heterologous systems (Hoffmann et al. 2021; Meadows et al. 2022). SIX3 and SIX6 are highly homologous, and we hypothesized that SIX6 would have similar promoter activity on clock genes and neuropeptides as SIX3. *Six6* overexpression significantly increased expression of the *Bmal1* luciferase reporter (Mann–Whitney Test, *U* = 0, *p* = 0.002; Figure 2a), which expresses the region ~1 kb upstream of *Bmal1*, an in‐frame coding region and ~1 kb of the *Bmal1* 3′ UTR (Brown et al. 2005). The *Per2* promoter expresses −1128 to +2129 of the promoter/enhancer region (Yoo et al. 2005), and was significantly repressed after 24 h (Mann–Whitney test, *U* = 6, *p* = 0.005; Figure 2b). We also found that *Six6* significantly increased *Vip* reporter activity (Mann–Whitney test, *U* = 12, *p* = 0.038; Figure 2c), which reflects a 1 kb promoter region upstream of *Vip* (Hatori et al. 2014). *Six6* did not significantly regulate a 5.5 kb region of the rat *Avp* promoter (Mann–Whitney test, *U* = 15, *p* = 0.083; Figure 2d) (Shapiro 2000). Together, these data demonstrate that, like SIX3, SIX6 can regulate the promoters of genes critical for SCN function.

### 3.3 Loss of *Six3* or *Six6* From NMS‐Expressing Neurons Does Not Impair Fertility in Females

**FIGURE 3:**
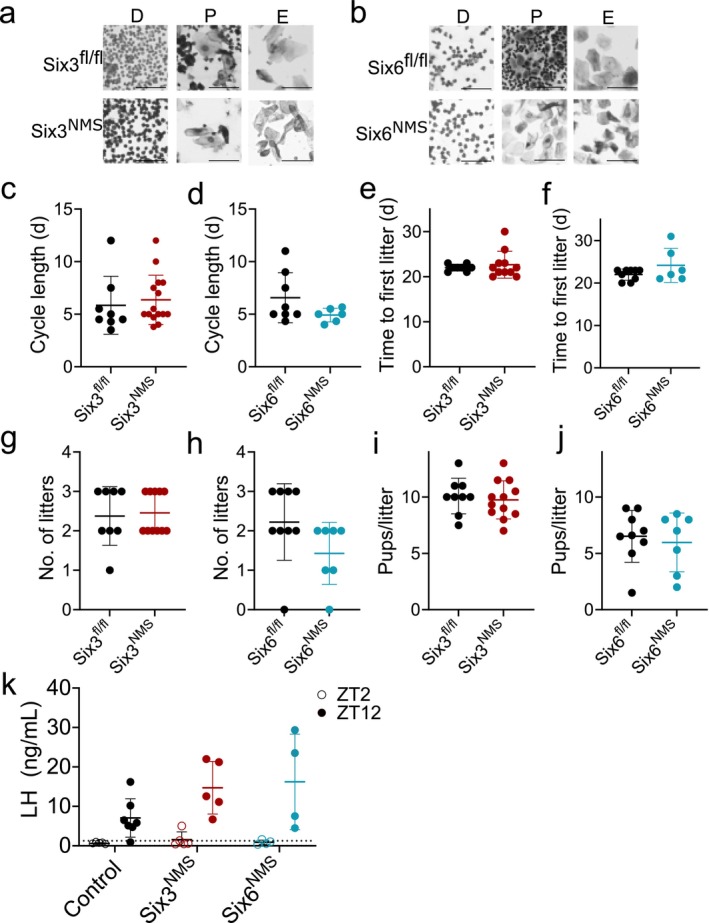
Neither *Six3* ^*NMS*^ nor *Six6* ^*NMS*^ female mice show changes in fertility measures. Representative vaginal smears for (a) *Six3* ^*fl/fl*^ and *Six3* ^*NMS*^ and (b) *Six6* ^*fl/fl*^ and *Six6* ^*NMS*^ females in diestrus (D), proestrus (P), and estrus (E). (c) Estrous cycle length for *Six3* ^*fl/fl*^ and *Six3* ^*NMS*^ (*n* = 8–15) and (d) *Six6* ^*fl/fl*^ and *Six6* ^*NMS*^ (*n* = 6–8) females. (e) Time to first litter for *Six3* ^*fl/fl*^ and *Six3* ^*NMS*^ (*n* = 8–11) and (f) *Six6* ^*fl/fl*^ and *Six6* ^*NMS*^ (*n* = 6–8) females. (g) Number of litters in 76 days for *Six3* ^*fl/fl*^ and *Six3* ^*NMS*^ (*n* = 6–8) and (h) *Six6* ^*fl/fl*^ and *Six6* ^*NMS*^ females (*n* = 7–9). (i) Pups per litter in *Six3* ^*fl/fl*^ and *Six3* ^*NMS*^ (*n* = 9–12) and (j) *Six6* ^*fl/fl*^ and *Six6* ^*NMS*^ females (*n* = 7–9). Data were analyzed using unpaired or Welch's *t*‐test. (k) LH levels at ZT 2 and ZT 12 during an induced preovulatory surge (control group is composed of both *Six3* ^*fl/fl*^ and *Six6* ^*fl/fl*^; *n* = 4–8). Data analyzed by two‐way ANOVA. Scale bar = 50 μm. Data expressed as mean ± SD.

Luteinizing hormone (LH) is vital for proper pubertal development, ovulation, and maintenance of fertility. NMS has previously been shown to interact with LH in rats (Vigo et al. 2007), and *Six3* ^*fl/fl*^ *‐Synapsin* ^*cre*^ females have reduced LH and delayed puberty (Hoffmann et al. 2021). To determine how loss of *Six3* and *Six6* in NMS neurons affects fertility, we assessed female age at vaginal opening (Table 2), estrous cycle length (Figure 3a–d), time to first litter (Figure 3e,f), number of litters in 76 days (Figure 3g,h), and pups per litter (Figure 3i,j). We found no differences between *Six3* ^*NMS*^ females and *Six3* ^*fl/fl*^ controls in estrous cycle length (unpaired *t*‐test, *t*(21) = 0.474, *p* = 0.640), time to first litter (Welch's *t*‐test, *t*(11.72) = 0.680, *p* = 0.510), number of litters (unpaired *t*‐test, *t*(17) = 0.275, *p* = 0.787), or pups per litter (unpaired *t*‐test, *t*(18) = 0.477, *p* = 0.642). Similarly, we found no difference between *Six6* ^*NMS*^ females and *Six6* ^*fl/fl*^ controls in estrous cycle length (Welch's *t*‐test, *t*(8.338) = 1.867, *p* = 0.097), time to first litter (Welch's *t*‐test, *t*(5.729) = 1.275, *p* = 0.252), number of litters (unpaired *t*‐test, *t*(14) = 1.755, *p* = 0.101), or pups per litter (unpaired *t*‐test, *t*(14) = 0.439, *p* = 0.667).

**TABLE 2:** *Six3* and *Six6* in NMS neurons have no effect on puberty onset.

	Six6^fl/fl^	Six6^NMS^	*n*, *p*, *t*	Six3 ^fl/fl^	Six3^NMS^	*n*, *p*, *t*
*Female*						
Age of vaginal opening (days)	29.27 ± 3.44	29.43 ± 3.15	*n* = 11, 7 *p* = 0.924 *t*(16) = 0.097	30.93 ± 3.15	30.64 ± 2.54	*n* = 14, 11 *p* = 0.805 *t*(23) = 0.250
Weight at vaginal opening (g)	12.52 ± 1.48	13.11 ± 0.98	*n* = 11, 7 *p* = 0.363 *t*(16) = 0.936	14.51 ± 1.70	14.87 ± 1.23	*n* = 14, 11 *p* = 0.554 *t*(23) = 0.600
*Male*						
Age of preputial separation (days)	28.50 ± 1.08	28.38 ± 1.80	*n* = 10, 13 *p* = 0.860 *t*(21) = 0.179	27.33 ± 1.03	26.50 ± 0.85	*n* = 6, 14 *p* = 0.076 *t*(18) = 1.881
Weight at preputial separation (g)	13.80 ± 2.34	13.11 ± 1.21	*n* = 10, 13 *p* = 0.360 *t*(21) = 0.936	15.72 ± 1.88	14.92 ± 1.78	*n* = 6, 14 *p* = 0.379 *t*(18) = 0.902

*Note:* Data presented as mean ± SD.

We then explored whether loss of *Six3* or *Six6* in NMS neurons could disrupt the timing of the preovulatory LH surge, which is regulated by the SCN. Using an induced surge model in ovariectomized mice, mice were sacrificed during the morning (ZT 2) or at lights off (ZT 12, expected time of LH surge). The surge threshold was the average of the AM control LH values plus three standard deviations (Figure 3k). *Six3* ^*fl/fl*^ and *Six6* ^*fl/fl*^ mice were combined for the control group. We found that 6 of 7 control mice exhibited a PM surge, along with all of the *Six6* ^*NMS*^ (4/4) and *Six3* ^*NMS*^ (5/5) mice. LH levels indicated a significant effect of time (as expected), but not of genotype (two‐way ANOVA, time *F*(1, 24) = 30.36, *p* < 0.001, genotype *F*(2, 24) = 2.223, *p* = 0.130, interaction *F*(2, 24) = 1.695, *p* = 0.205). Therefore, the ability to mount a normally timed LH surge is unaffected by the loss of either *Six3* or *Six6* in NMS neurons in LD conditions.

### 3.4 Loss of *Six3* From NMS Cells Results in Reduced Sperm Motility in Conditional Knock‐Out Males

**FIGURE 4:**
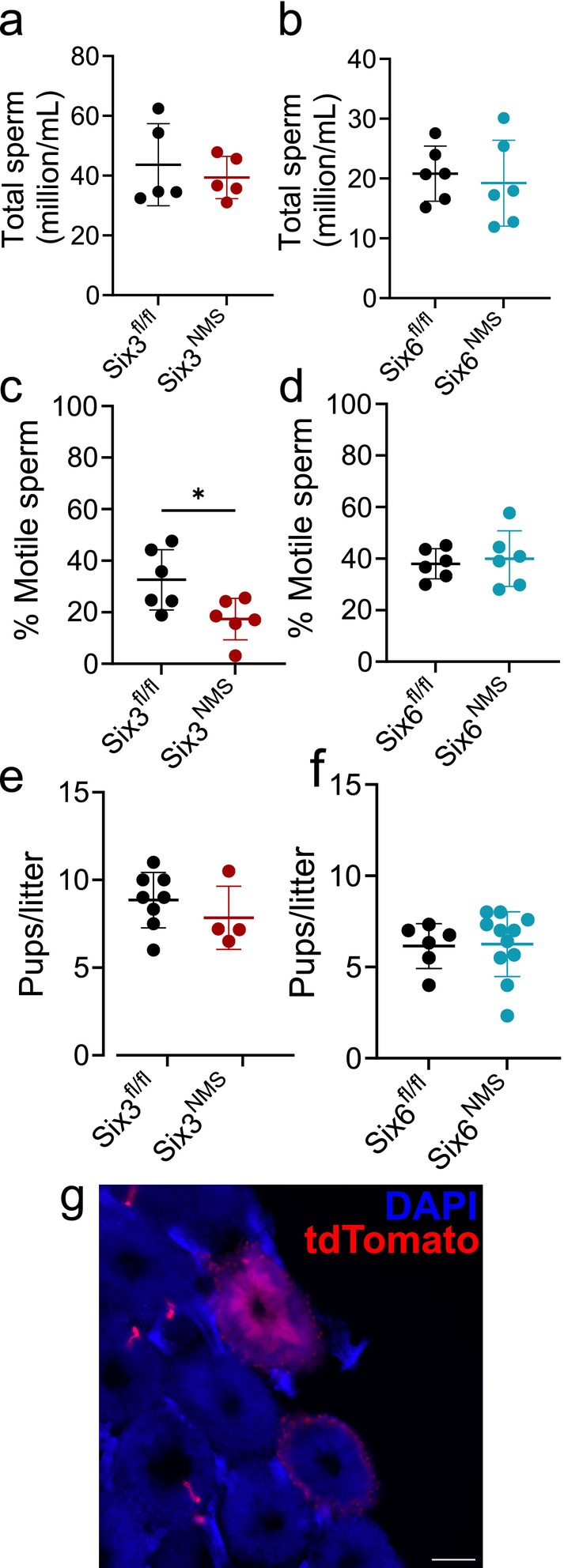
*Six3*, but not *Six6*, in NMS cells affects sperm through an unknown mechanism. (a) Total sperm count for *Six3* ^*fl/fl*^ and *Six3* ^*NMS*^ (*n* = 5) and (b) *Six6* ^*fl/fl*^ and *Six6* ^*NMS*^ males (*n* = 6). (c) Percent motile sperm for *Six3* ^*fl/fl*^ and *Six3* ^*NMS*^ (*n* = 5) and (d) *Six6* ^*fl/fl*^ and *Six6* ^*NMS*^ (*n* = 6) males. (e) Pups per litter for *Six3* ^*fl/fl*^ and *Six3* ^*NMS*^ (*n* = 4–8) and (f) *Six6* ^*fl/fl*^ and *Six6* ^*NMS*^ (*n* = 6–11). (g) Representative image from an adult mouse testis with *Nms* ^*cre*^ reporter expression (tdTomato, red) counterstained with DAPI (blue). Scale bar = 100 μm. Data were analyzed using unpaired *t*‐test. Data expressed as mean ± SD. Significance indicated by *p < 0.05.

To determine whether loss of *Six3* or *Six6* affects the hypothalamic–pituitary‐gonadal axis in males, we first observed pubertal onset in both *Six3* ^*NMS*^ and *Six6* ^*NMS*^ male mice. No differences were found in the weight or age at preputial separation between either *Six3* ^*NMS*^ or *Six6* ^*NMS*^ mice and respective flox/flox controls (Table 2). After sexual maturity, we measured total sperm (Figure 4a,b), and the percent motile sperm (Figure 4c,d) in both *Six3* ^*NMS*^ and *Six6* ^*NMS*^ male mice. *Six3* ^*NMS*^ males showed a reduction in the percent of motile sperm (unpaired *t*‐test, *t*(10) = 2.632, *p* = 0.025). We measured plugging efficiency in *Six3* ^*NMS*^ mice by pairing them with wild‐type females and measuring the time to plug and found no difference compared to control *Six* ^*fl/fl*^ males (2.38 ± 0.59 vs. 2.87 ± 0.44 days, unpaired *t*‐test, *t*(14) = 0.675, *p* = 0.511). Then, we paired male *Six3* ^*NMS*^ or *Six6* ^*NMS*^ with wild‐type females and examined the number of pups per litter (Figure 4e,f) and found no differences in number from either line (unpaired *t*‐test, *t*(10) = 1.001, *p* = 0.340 and *t*(15) = 0.128, *p* = 0.900, respectively).

A possible mechanism for reduced sperm motility in *Six3* ^*NMS*^ is a local testis effect. NMS has previously been reported in the testis of rats (Ida et al. 2005), and *Six3* is expressed in spermatids and spermatocytes (Lukassen et al. 2018b, 2018c). We examined whether *Nms* ^*cre*^ was expressed in the test is using a tdTomato^NMS^ reporter mouse. In mature males, we found evidence of sporadic tdTomato expression, raising the possibility that a local testis knockdown of *Six3* may drive the phenotype (Figure 4g).

### 3.5 Loss of *Six3* in NMS Neurons Does Not Affect the Circadian Rhythm of Corticosterone

**FIGURE 5:**
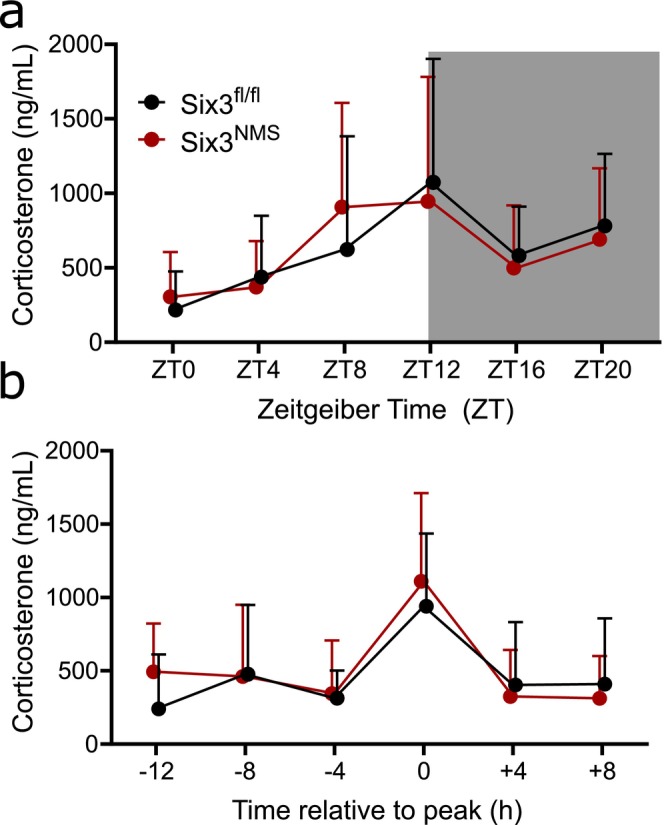
Loss of *Six3* in NMS neurons does not affect corticosterone rhythms. Measurements from serial tail bleeds in male and female mice showed no differences in peak amplitude and timing between control *Six3* ^*fl/fl*^ and *Six3* ^*NMS*^ mice in (a) 12:12 light:dark conditions (*n* = 16–17; 9 males, 8 females) and (b) constant darkness aligned by peak time (*n* = 14–17; 9 males, 5–8 females). Shaded area in (a) indicates times of lights off. Data were analyzed by two‐way ANOVA. Data expressed as mean ± SD.

We monitored corticosterone rhythm in *Six3* ^*NMS*^ mice to determine if loss of *Six3* in NMS‐containing neurons impacted the circadian rhythm of corticosterone. After habituation, male and female mice in 12 h light:12 h dark (LD) conditions were tail bled every 4 h for 24 h. Using a two‐way ANOVA analysis, corticosterone levels showed a significant effect of time in both groups, and no effect of genotype (two‐way repeated measures ANOVA, effect of time *F*(5, 155) = 10.110, *p* < 0.001; genotype *F*(1, 31) = 0.000066, *p* = 0.994; interaction *F*(5, 155) = 0.855, *p* = 0.513; Figure 5a). Mice were then allowed to free run for 2 weeks in constant dark (DD) conditions before undergoing tail bleeds again. Sampling times were not aligned to circadian activity, and due to drifts in the corticosterone peak due to individual differences in free running rhythms, the data are shown as aligned to the peak cortisol time (Figure 5b). Similar to the LD conditions, we found a significant effect of time and no effect of genotype (mixed‐effects analysis, time *F*(3.33, 95.91) = 28.74, *p* < 0.001; genotype *F*(1, 29) = 0.1668, *p* = 0.686; interaction *F*(3.33, 95.91) = 2.049, *p* = 0.106). Therefore, *Six3* in NMS neurons does not contribute to the amplitude or timing of the corticosterone surge.

### 3.6 Loss of *Six3*, but Not *Six6*, From NMS Neurons Shortens the Free‐Running Circadian Period

**FIGURE 6:**
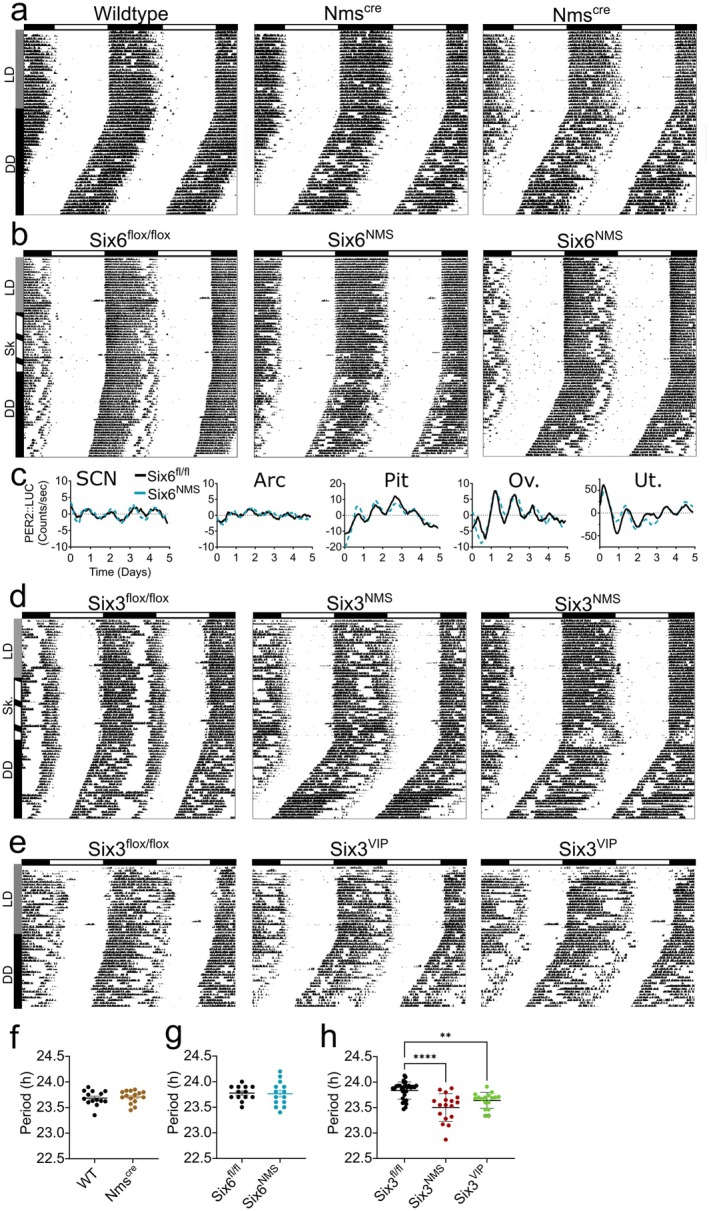
*Six3*, but not *Six6* in NMS neurons, regulates the free‐running locomotor rhythm. (a) Representative actograms during light–dark and constant darkness from (a) one *Nms* ^*cre*^ negative and two representative *Nms* ^*cre*^ positive mice and (b) one control *Six6* ^*fl/fl*^ and two *Six6* ^*NMS*^ mice. (c) Representative PER2::LUC recordings from SCN, arcuate nucleus (Arc), pituitary (pit), ovary (ov) and uterus (ut) from *Six6* ^*fl/fl*^ *PER2::LUC* and *Six6* ^*NMS*^ *PER2::LUC* mice. Representative actograms for (d) one control *Six3* ^*fl/fl*^ control and two *Six3* ^*NMS*^ mice and (e) one control *Six3* ^*fl/fl*^ control and two *Six3* ^*VIP*^ mice. (f) The free‐running period (tau) is not affected by the presence of the Cre allele compared to WT littermates (*n* = 14–15; 5–6 males, 8–9 females). (g) *Six6* ^*NMS*^ have normal free‐running periods compared to *Six6* ^*fl/fl*^ controls (*n* = 16; 4 males, 4 females). (h) *Six3* ^*NMS*^ and *Six3* ^*VIP*^ have significantly shorter free‐running periods than *Six3* ^*fl/fl*^ control mice (*n* = 14–23, 9–15 males, 8–15 females). Data were analyzed using unpaired *t*‐test or one‐way ANOVA, Sidack's posthoc test. Data expressed as mean ± SD. Significance indicated by **p < 0.01, ****p < 0.0001. LD: 12:12 Light:Dark; Sk: 1:10:1:12 skeleton photoperiod; DD: Constant darkness. Arc, arcuate nucleus; Ov., ovary; Pit, pituitary; Ut., uterus.

To determine how SCN function was altered in *Six3* ^*NMS*^ and *Six6* ^*NMS*^ mice, we measured wheel running locomotor activity. First, we wanted to confirm that the presence of the *Nms* ^*cre*^ allele alone had no effect on locomotor activity. We found that the free‐running rhythm was unaffected by the *Nms* ^*cre*^ allele (Table 3; Figure 6a,f; unpaired *t*‐test, *t*(27) = 0.426, *p* = 0.674). We also evaluated circadian amplitude, onsets precision, phase angle, and alpha, and found no effect by unpaired *t*‐test (Table 4). After establishing no effect of the *Nms* ^*cre*^ allele itself, we then asked whether conditional knockout of *Six6* in NMS neurons (*Six6* ^*NMS*^) would affect locomotor activity compared to littermate *Six6* ^*fl/fl*^ controls. We found no significant difference in the free running period (Figure 6b,g; unpaired *t*‐test, *t*(25) = 0.166, *p* = 0.869) or other locomotor behavior (Table 3). We confirmed this lack of circadian change with ex vivo explants from the SCN, arcuate nucleus, pituitary gland, ovaries, and uterus, which showed no significant differences in the period of the PER2::LUC rhythms between *PER2::LUC*‐*Six6* ^*NMS*^ mice and controls (Figure 6c; Table 4).

**TABLE 3:** Circadian wheel running parameters.

	Wild‐type (*n* = 14)	NMS^cre^ (*n* = 14)	*t*‐test statistics	Six6^flox/flox^ (*n* = 8)	Six6^NMS^ (*n* = 8)	*t*‐test statistics	Six3^flox/flox^ (*n* = 23)	Six3^NMS^ (*n* = 16)	Six3^VIP^ (*n* = 14)	One‐way ANOVA
*LD measures*										
X^2^ amplitude	2089 ± 383	1917 ± 468	*t*(26) = 1.063, *p* = 0.297	3520 ± 479	3465 ± 915	*t*(14) = 0.151, *p* = 0.882	2074 ± 572	2328 ± 615	2019 ± 321	*F*(2, 50) = 1.528, *p* = 0.227
Onset precision	0.35 ± 0.37	0.18 ± 0.26	*t*(26) = 1.376, *p* = 0.181	0.11 ± 0.05	0.14 ± 0.013	*t*(14) = −0.548, *p* = 0.593	0.37 ± 0.18	0.32 ± 0.18	0.29 ± 0.13	*F*(2, 50) = 1.015, *p* = 0.370
Phase angle	0.24 ± 0.32	0.17 ± 0.14	*t*(26) = 0.752, *p* = 0.459	0.26 ± 0.09	0.20 ± 0.13	*t*(14) = 1.081, *p* = 0.298	0.14 ± 0.47	0.10 ± 0.35	0.14 ± 0.21	*F*(2, 50) = 0.071, *p* = 0.932
*DD measures*										
X^2^ amplitude	1779 ± 370	1674 ± 410	*t*(26) = 0.709, *p* = 0.485	4652 ± 1560	4357 ± 1220	*t*(14) = 0.421, *p* = 0.680	2664 ± 704	2698 ± 1050	2308 ± 636	*F*(2, 50) = 1.084, *p* = 0.346
Onset precision	0.42 ± 0.18	0.47 ± 0.2	*t*(26) = −0.253, *p* = 0.802	0.41 ± 0.21	0.30 ± 0.14	*t*(14) = 1.298, *p* = 0.215	0.55 ± 0.23	0.48 ± 0.24	0.48 ± 0.24	*F*(2, 50) = 1.674, *p* = 0.198
Alpha (h)	13.26 ± 1.94	13.93 ± 1.55	*t*(26) = −0.999, *p* = 0.327	13.55 ± 1.52	13.78 ± 1.86	*t*(14) = −0.272, *p* = 0.790	16.13 ± 1.77	16.6 ± 2.1	15.38 ± 3.2	*F*(2, 50) = 1.092, *p* = 0.343

*Note:* Data presented as mean ± SD.

**TABLE 4:** Periods of proestrus female ex vivo explants period as recorded via lumicycle. ZT3‐4.

	Control	Six6^NMS^	*t*‐test statistics
Female SCN (*n* = 5)	26.61 ± 1.95	25.47 ± 0.63	*t*(8) = 1.249, *p* = 0.247
Female arcuate (*n* = 5–6)	25.10 ± 1.56	24.21 ± 1.00	*t*(9) = 1.092, *p* = 0.303
Female pituitary (*n* = 5–6)	24.90 ± 0.48	25.50 ± 0.87	*t*(9) = 1.443, *p* = 0.183
Ovary (*n* = 6)	25.36 ± 0.75	25.59 ± 1.14	*t*(10) = 0.410, *p* = 0.691
Uterus (*n* = 5)	25.34 ± 0.85	26.00 ± 1.00	*t*(8) = 1.128, *p* = 0.292

*Note:* Data presented as mean ± SD.

Finally, we wanted to examine the effect of conditional loss of *Six3* in SCN populations. We measured locomotor activity in both *Six3* ^*NMS*^ mice as well as *Six3* ^*fl/fl*^ x *Vip* ^*cre*^ mice (*Six3* ^*VIP*^) neurons, given the extensive overlap of VIP and NMS (Lee et al. 2015). We found that there was a significant difference in the free running rhythm between *Six3* ^*fl/fl*^, *Six3* ^*NMS*^, and *Six3* ^*VIP*^ (Figure 6d,g; one‐way ANOVA (*F*(2, 62) = 16.34, *p* < 0.001)). Post hoc analysis indicated that both *Six3* ^*NMS*^ and *Six3* ^*VIP*^ were significantly different from *Six3* ^*fl/fl*^ controls (Šidák's multiple comparisons test, *p* < 0.001 and *p* = 0.004, respectively). There were no other differences by one‐way ANOVA among the other measures (Table 3).

### 3.7 Loss of *Six3* From NMS Neurons Does Not Affect *Avp, Vip* or *Nms* Cell Count in the SCN

**FIGURE 7:**
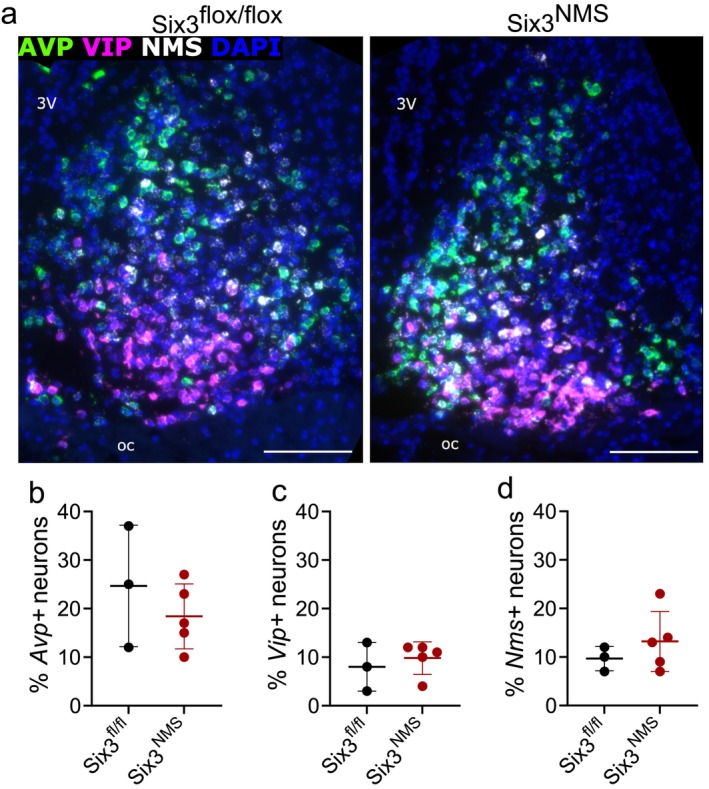
Loss of *Six3* in NMS neurons does not alter the number of *Avp* or *Vip* ‐containing cells in the SCN. (a) Representative images of RNAScope for *Avp* (green), *Vip* (magenta), and *Nms* (white) with DAPI (blue) counterstain in *Six3* ^*fl/fl*^ (left) and *Six3* ^*NMS*^ (right) SCN. (b) Quantification of *Avp*‐containing cells shows no difference between *Six3* ^*fl/fl*^ and *Six3* ^*NMS*^ (*n* = 3–5). (c) Quantification of *Vip*‐containing cells shows no difference between *Six3* ^*fl/fl*^ and *Six3* ^*NMS*^ (*n* = 3–5). (d) Quantification of *Nms*‐containing cells shows no difference between *Six3* ^*fl/fl*^ and *Six3* ^*NMS*^ (*n* = 3–5). Data were analyzed using unpaired *t*‐test. Scale bar = 100 μm. Data expressed as mean ± SD. 3V, third ventricle; OC, optic chiasm.

To probe a potential mechanism for the accelerated free running rhythm of *Six3* ^*NMS*^ mice, we hypothesized that these animals may have an alteration in SCN peptides. We performed RNAscope for *Avp, Vip*, and *Nms* in the SCN of mice harvested from ZT 8–11 (Figure 7a, *n* = 3–5). We found that there was no significant difference in the percentage of neurons that express *Vip* between *Six3* ^*fl/fl*^ and *Six3* ^*NMS*^ mice (unpaired *t*‐test, *t*(6) = 0.620, *p* = 0.558; Figure 7b), *Avp* (unpaired *t*‐test, *t*(6) = 0.948, *p* = 0.380; Figure 7c), or *Nms* (unpaired *t*‐test, *t*(6) = 0.921, *p* = 0.392; Figure 7d).

## 4 Discussion

Despite their high homology and overlapping expression, our data suggest that *Six3* and *Six6* have distinct roles in regulating circadian rhythms in the post‐proliferative SCN. Using published single cel RNASeq datasets, we found that approximately half of *Nms‐*containing neurons express both *Six3* and *Six6* (Steuernagel et al. 2022). Given the shortened free‐running period in locomotor activity in *Six3* ^*NMS*^ but not *Six6* ^*NMS*^ animals, this overlap suggests distinct roles of *Six3* and *Six6* within the same population of NMS neurons; *Six3* contributes to the pace of SCN function in the adult SCN, whereas *Six6* is dispensable.

We cannot rule out a difference in knockout efficiency between the *Six3* and *Six6* flox alleles. Due to the high homology between SIX3 and SIX6, we were unable to find working antibodies that would allow us to distinguish between the two proteins. Our previous attempts to use a custom RNAscope probe to quantify *Six3* signal produced an atypical fluorescent signal in neurons with the conditional knockout. This abnormal signal is likely an artifact of recombination (Lavalle et al. 2022). We and others have observed robust circadian and reproductive phenotypes with conditional knockout using the Six3^fl/fl^ (Hoffmann et al. 2021; Lavalle et al. 2022; Liu et al. 2006; Pandolfi et al. 2018; Yu et al. 2024) and the Six6^fl/fl^ lines (Hoffmann et al. 2019; Pandolfi, Breuer, et al. 2019; Pandolfi, Tonsfeldt, et al. 2019). Similarly, *Nms* ^*cre*^ is well‐characterized and has been used extensively by many groups for conditional knockout with phenotypic effect (Asano et al. 2023; Bussi et al. 2023; Lee et al. 2015; Tonsfeldt et al. 2022; Yurgel et al. 2025).

*Six3* is already robustly expressed in the progenitor domain and eventual SCN at E10.5, and persists until adulthood; *Six6* expression in the region begins at E11.5 and similarly persists (VanDunk et al. 2011). We surmise, then, that recombination of *Six3* and *Six6* occurs within the same time period in both models because they are both expressed prior to the onset of *Nms* ^*cre*^. We demonstrate here that there is no *Nms* ^*cre*^‐mediated recombination in the SCN by E16.5, while *Nms* expression occurs in the SCN by P0–2 (Cheng et al. 2021; Van Loh et al. 2023); thus, recombination of *Six3* and *Six6* likely occurs in this 4‐day window after SCN neurogenesis but prior to neuronal pruning initiation.

The absence of a locomotor phenotype in the *Six6* ^*NMS*^ mice suggests that despite continued expression of *Six6* into adulthood, there is a more essential role for *Six6* in SCN development. Full body *Six6* ^*−/−*^ mice display highly variable optic nerve formation, and occasionally no identifiable SCN (Clark et al. 2013). However, the subset of *Six6* ^−/−^ mice with optic nerves has relatively normal rhythmicity in LD, despite the absence of detectable SCN markers AVP or VIP (Clark et al. 2013). Using a *GnRH* ^*cre*^ allele with ectopic SCN expression, loss of *Six6* in these neurons results in failure to develop an SCN and abnormal locomotor rhythms (Pandolfi, Breuer, et al. 2019). The *GnRH* ^*cre*^ allele is turned on by E11.5 (Pandolfi, Breuer, et al. 2019), indicating an early developmental period of *Six6* action. In our model, when recombination occurs after E16, *Six3* may be able to compensate for the loss of *Six6* in *Six6* ^*NMS*^ mice, resulting in normal SCN development and locomotor rhythms.

Our in vitro data showed variations in the transcriptional actions of *Six6* among the genes we tested. Our *Six6* overexpression assays were performed using the same cells and same reporter vectors in the same lab as the *Six3* in vitro studies, allowing us to compare our findings to previously published data of *Six3*. We found that SIX3 and SIX6 can both regulate *Vip, Per2*, and *Bmal1* (Meadows et al. 2022). SIX6 did not regulate *Avp* in this context, while SIX3 was able to regulate the *Avp* promoter (Meadows et al. 2022). *Six6* was not identified as being rhythmic in a recent single cell RNASeq study of the SCN, and *Six3* rhythmicity was only detected in a non‐NMS‐expressing SCN neuron subtype (Wen et al. 2020); therefore, complementary temporal organization of the two transcription factors seems unlikely. A limitation of these results is that we are investigating putative promoter regions in a heterologous system; while SIX6 has the capacity to regulate *Bmal1, Per2*, and *Vip* in our overexpression system in vitro, our behavioral data suggests that loss of *Six6* promoter activity is not functionally significant in the control of SCN‐regulated locomotor activity or reproductive parameters, at least in NMS neurons. It is possible that given sequence homology, *Six3* and *Six6* can compensate for one another. Future work could pursue whether *Six3* and *Six6* bind clock targets in vivo and whether loss of both genes together in NMS neurons would produce more profound phenotypic outcomes.

SIX3 and SIX6 show a high degree of overlap in structure, function, and expression. Due to this overlap, SIX3 and SIX6 are grouped together in the SIX family genes, exhibiting both structural and functional differences from the rest of the SIX family, which are not expressed in the developing hypothalamus (Kawakami et al. 2000; Meurer et al. 2021). Like the SCN, SIX3 and SIX6 also overlap in retinal progenitor cells where they have distinct roles (Ferrena et al. 2024; Raven et al. 2018). In the retinal cell populations, loss of either *Six3* or *Six6* is not sufficient to affect neuroretinal progenitor specification, but somatic loss of *Six6* with *Six3* knockdown arrests the specification. As observed by these authors, it is unclear if functional compensation by either *Six3* or *Six6* causes the absence of a phenotype in the *Six6* or *Six3* knockouts respectively, or if the genes have different targets (Raven et al. 2018). In our case, functional compensation by *Six3* for *Six6* may underly the absence of a phenotype in *Six6* ^*NMS*^ mice, but *Six6* is unable to compensate for *Six3* in *Six3* ^*NMS*^. It would be interesting to determine if combined loss of *Six3* and *Six6* in NMS neurons has a more severe locomotor phenotype than *Six3* ^*NMS*^ alone.

In our model, we found no *Nms* ^*cre*^ recombination at E16.5 in the SCN, a timepoint just past the critical window of SCN neurogenesis (Kabrita and Davis 2008; Shimada and Nakamura 1973; VanDunk et al. 2011). Using *Nms* ^*cre*^ to knock down *Six3* later in development resulted in phenotypes distinct from those in the full‐body deletion of either *Six3* ^+/−^ or the post‐proliferative *Six3* ^*fl/fl*^ *‐Synapsin* ^*cre*^. Previous work has found that deletion of *Six3* in post‐proliferative neurons (driven by *Synapsin* ^*cre*^) results in disorganized and weaker locomotor rhythms in males and females as measured by *χ* ^2^ amplitude (Hoffmann et al. 2021; Meadows et al. 2022). However, we did not find that loss of *Six3* in NMS neurons caused a similar disorganization. In contrast, *Six3* ^*fl/fl*^ *‐Synapsin* ^*cre*^ mice had no changes to tau, whereas the *Six3* ^*NMS*^ mice had shorter free running periods. Difference in location and timing of *Six3* recombination likely drives these differences. *Synapsin* ^*cre*^ targets a larger population of neurons than *NMS* ^*cre*^, including neurons beyond the SCN. *Synapsin* ^*cre*^ also turns on earlier in development, showing recombination in the ventral hypothalamus at E15.5 (although this is not localized to the SCN) (Perry et al. 2022). By postnatal Day 7, there is substantial *Synapsin* ^*cre*^‐mediated recombination throughout the brain (Perry et al. 2022). Furthermore, the presence of the *Synapsin* ^*cre*^ allele has recently been reported to affect visual acuity and sex‐specific behavioral assays (Baghdadi et al. 2023), which may also affect the interpretations of the *Six3* ^*fl/fl*^ *‐Synapsin* ^*cre*^ data. The contrast in behavior between the two cre alleles is compelling, and further work will be needed to identify whether it is the timing or neural population of *Six3* recombination driving the different effects.

Changes to the circadian system can impact male reproduction and sperm, as seen in altered feeding paradigms and shift work (Kohn and Pastuszak 2017; Swamy et al. 2018). Our work demonstrates that sperm motility can be mildly impacted by the loss of *Six3*, but not *Six6*, from NMS cells. Conditional knockout of *Six3* from kisspeptin neurons, which stimulate GnRH release, also reduces sperm motility (Lavalle et al. 2022). Recent work has found that NMS neurons from the SCN project directly to kisspeptin neurons in the anteroventral periventricular nucleus (Abdulmajeed et al. 2024), although the role of this population in males is unclear. *Nms* expression is dependent on steroid status, and NMS administration increases LH in female mice and male pigs (Jin et al. 2019; Mori et al. 2008; Vigo et al. 2007). High LH in males, in turn, decreases sperm quality and motility (Meeker et al. 2007), which may provide a circuit through which loss of *Six3* from NMS neurons impacts sperm motility. However, we also detected *Nms* ^*cre*^ ‐mediated recombination in the testis. RNASeq analysis indicates that certain subpopulations in the adult mouse testis, including round spermatids and spermatocytes, express *Six3* (see Data Citation 1; Lukassen et al. 2018a, 2018b), so a local effect cannot be ruled out. The reduction in sperm motility did not affect male fertility.

Female fertility was unaffected in *Six3* ^*NMS*^ and *Six6* ^*NMS*^ mice. Female *Six3* ^+/−^ and *Six6* ^*−/−*^ mice are subfertile, likely due to abnormal migration and reduced numbers of GnRH neurons (Larder et al. 2011; Pandolfi et al. 2018). While conditional deletion of *Six6* in GnRH neurons similarly decreased GnRH neurons (Pandolfi, Tonsfeldt, et al. 2019), conditional deletion of *Six3* in GnRH neurons increased GnRH neurons and restored fertility (Pandolfi et al. 2018), suggesting the effects of *Six3* on reproduction are not mediated by GnRH neurons. In further support, the *Synapsin* ^*cre*^ spares GnRH neurons, but *Six3* ^*fl/fl*^‐*Synapsin* ^*cre*^ female mice have a disrupted preovulatory LH surge and subfertility. However, due to the widespread expression of Cre in the *Synapsin* ^*cre*^ mouse, this effect cannot be specifically localized to the SCN (Hoffmann et al. 2021). Both *Six3* ^*NMS*^ and *Six6* ^*NMS*^ failed to recapitulate the subfertility phenotypes of other *Six3* or *Six6* knockouts or knockdowns, indicating that these genes are not regulating reproduction at the level of the NMS neurons.

Mice that have NMS neurons selectively silenced in the SCN using tetanus light chain toxin lose rhythmic activity (Lee et al. 2015), but full‐body NMS knockout mice show no differences in rhythmic activity (Malendowicz and Rucinski 2021). The majority of AVP and VIP SCN neurons also express NMS (Lee et al. 2015). We found that *Six3* ^*NMS*^ mice did not have a difference in the number of *Avp, Vip, or Nms*‐containing neurons, suggesting that the loss of *Six3* does not affect production of these peptides in vivo. This was somewhat surprising, as we saw a similar decrease in the locomotor period of the *Six3* ^*NMS*^ mice as *Six3* ^*VIP*^ mice, suggesting that the effects seen in *Six3* ^*NMS*^ mice may be due to *Six3* action in VIP neurons; in addition, decreased VIP shortens locomotor period (Colwell et al. 2003; Joye et al. 2020). We only assayed the SCN peptides at one time point (ZT 8–11) when *Avp* is predicted to be high and *Vip* levels rising (Dardente et al. 2004); with this approach, we would not observe differences during other time points that may implicate *Vip*. Therefore, it appears that the mechanism driving the changes in locomotor period is more subtle than peptide percentages. One potential mechanism is that loss of *Six3* in NMS neurons may shorten circadian rhythms by promoting the molecular clock components required for daily oscillations instead of altering peptides. Mice in which clock genes are conditionally altered in NMS neurons either through a deletion of *Bmal1* or through expression of a dominant‐negative form of *Clock* (Clock‐Δ19) (Lee et al. 2015; Tonsfeldt et al. 2022) have disrupted rhythms in constant darkness.

We found that *Six3* and *Six6* have differing roles in NMS neurons in the regulation of circadian rhythms in the adult animal. Loss of *Six3* from NMS neurons, which occurs after SCN neurogenesis, results in a mild reduction of sperm in males and a shortened free‐running period in both males and females. However, loss of *Six6* from NMS neurons results in no reproductive or circadian phenotypes. Together, these data show that *Six3* and *Six6*, though related and often compensatory genes, play different functions in the SCN after its development.

## Author Contributions

**Pamela L. Mellon, Lauren E. Chun, Hanne M. Hoffmann, and Karen J. Tonsfeldt:** conceptualization. **Pamela L. Mellon, Lauren E. Chun, Hanne M. Hoffmann, and Karen J. Tonsfeldt:** methodology. **Brooke M. Van Loh, Geneva A. Dunn, Lauren E. Chun, Meera M. Patel, Nay Chi P. Naing, Duong Nguyen, Alexandra M. Yaw, Jessica Cassin, and Karen J. Tonsfeldt:** investigation. **Michael R. Gorman, Pamela L. Mellon:** resources. **Brooke M. Van Loh, Lauren E. Chun, and Karen J. Tonsfeldt:** writing – original draft. **Brooke M. Van Loh, Geneva A. Dunn, Lauren E. Chun, Alexandra M. Yaw, Jessica Cassin, Pamela L. Mellon, Hanne M. Hoffmann, and Karen J. Tonsfeldt:** writing – review and editing. **Brooke M. Van Loh, Geneva A. Dunn, and Karen J. Tonsfeldt:** visualization. **Pamela L. Mellon, Hanne M. Hoffmann, and Karen J. Tonsfeldt:** supervision. **Pamela L. Mellon, Hanne M. Hoffmann, and Lauren E. Chun:** funding acquisition.

## Funding

This work was supported by the National Institutes of Health (NIH) Grants R01 HD072754, R01 HD100580, and R01 HD082567 (to P.L.M.). It was also supported by NIH/Eunice Kennedy Shriver National Institute of Child Health and Human Development (NICHD) P50 HD012303 as part of the National Centers for Translational Research in Reproduction and Infertility (P.L.M.). P.L.M. was also partially supported by P30 DK063491, P30 CA023100, and P42 ES010337. B.M.V. was supported by T32 HD087166 and F31 HD114529. L.E.C. was supported by T32 HD007203 and F32 HD098805. N.C.P.N. was partially supported by McNair Research Program. A.M.Y. was partially supported by T32 HD087166, F32 HD107852, and K99 HD113843. J.C. was partially supported by T32 HD007203, K12 GM068524, and K99 HD107217. K.J.T. was partially supported by T32 HD007203, P42 ES010337, F32 HD090837, and K99 NS119291. H.M.H. was partially supported by K99/R00 HD084759 and the United States Department of Agriculture National Institute of Food and Agriculture Hatch project MICL1018024. The UCSD School of Medicine Microscopy core was supported by NINDS P30 NS047101. The funders had no role in study design, data collection and interpretation, or the decision to submit the work for publication.

## Conflicts of Interest

The authors declare no conflicts of interest.

## Supporting information


**Data S1:** jnr70123‐sup‐0001‐Supinfo.zip.

## Data Availability

The data that support the findings of this study are available from the corresponding author upon reasonable request.
