# Supplementary material for: The Transcription Factors Six3 and Six6 in Neuromedin‐S Neurons Differentially Affect Circadian Rhythms
Source: J Neurosci Res. 2026 Apr 5;104(4):e70123. doi: 10.1002/jnr.70123 (PMC13051014; doi:10.1002/jnr.70123)
Supplement: Supplementary file 1 — Data S1: jnr70123‐sup‐0001‐Supinfo.zip. [file rev-2-editor-1-recommendation.pdf › rev-2-editor-1-recommendation.pdf]

WILEY

Dear Dr. Larry S. Sherman,

Tamara Franklin has recommended a revision for "The transcription factors Six3 and Six6 in neuromedin-S neurons differentially affect circadian rhythms."
by Karen J. Tonsfeldt.

The handling editor provided the following comments:

> *Thank you for your careful response to the reviewers' comments. The reviewers have agreed that many of the points have been addressed and that the manuscript is much improved. However, there are still some revisions suggested. The majority of these are relatively minor but important edits (e.g., to improve statistical reporting and data presentation). Confirmation that Six3 or Six6 is knocked out by Nms-Cre in Six3-Nms or Six6-Nms mice has also been suggested, and this seems like an important consideration. Please address all comments fully.*

To review this recommendation and submit your input, please visit the manuscript details page.

MANUSCRIPT DETAILS

Kind regards,
Journal of Neuroscience Research

This email was sent to shermanl@ohsu.edu by John Wiley & Sons, Inc.
111 River Street, Hoboken, NJ 07030 USA. 877-762-2974.
For more information, please see our privacy policy.
