# Supplementary material for: The Transcription Factors Six3 and Six6 in Neuromedin‐S Neurons Differentially Affect Circadian Rhythms
Source: J Neurosci Res. 2026 Apr 5;104(4):e70123. doi: 10.1002/jnr.70123 (PMC13051014; doi:10.1002/jnr.70123)
Supplement: Supplementary file 1 — Data S1: jnr70123‐sup‐0001‐Supinfo.zip. [file rev-2-editor-decision-letter.pdf › rev-2-editor-decision-letter.pdf]

WILEY

Dear Dr. Karen J. Tonsfeldt,

Thank you for submitting your manuscript "The transcription factors Six3 and Six6 in neuromedin-S neurons differentially affect circadian rhythms." to Journal of Neuroscience Research.

After careful assessment, our editorial team has decided your manuscript requires minor revisions in order to proceed with the peer review process and be considered for publication. Reviewer comments are included at the bottom of this email to assist you in revising your manuscript.

We ask that you return your revised manuscript by March 21, 2026. If you need more time, please let us know. We are happy to discuss an extended deadline.
When submitting your revised manuscript, you will be asked to upload an author response letter that details the revisions you have made in response to editor and reviewer comments. You may also be required to show revisions made within the manuscript files. Please follow any journal-specific guidelines for formatting and submitting revisions outlined in the author guidelines page.

When you have finished revising your manuscript, follow the link below to submit your revision:

Submit your revised files

Thank you for submitting your work to Journal of Neuroscience Research. We look forward to receiving your revised manuscript.

Kind regards,
Larry S. Sherman
Journal of Neuroscience Research

Handling editor comment:

> *Thank you for your careful response to the reviewers' comments. The reviewers have agreed that many of the points have been addressed and that the manuscript is much improved. However, there are still some revisions suggested. The majority of these are relatively minor but important edits (e.g., to improve statistical reporting and data presentation). Confirmation that Six3 or Six6 is knocked out by Nms-Cre in Six3-Nms or Six6-Nms mice has also been suggested, and this seems like an important consideration. Please address all comments fully.*

The following reviewer comments were taken into consideration during the peer review process.

> ***Reviewer 1 Comments to the Author***
>
> *The authors have responded appropriately to the reviewer's comments. Please confirm only the following points before publication.*
>
> *Please clearly label what each panel's Y-axis represents.*
>
> ***Reviewer 2 Comments to the Author***
>
> *With regard to Comment #4, I requested an investigation into the extent to which Six3 or Six6 is knockedout by Nms-Cre in Six3-Nms or Six6-Nms mice, respectively; however, no experiments addressing this issue were performed. As demonstrated by the experiments shown in Figure 7, however, RNAScope experiments targeting Nms and either Six3 or Six6 should be feasible.*
>
> *With regard to Comment #6, I did not find any text marked for correction in red in lines 542–545. Does the updated citation refer to "Lukassen, S., Bosch, E., Ekici, A. B., & Winterpacht, A. (2018a). Cell and gene data for testicular single-cell RNA-Seq." (lines 770–771)? I am unsure whether this is a paper or a website. I could not find it through searching, so please could you provide specific details?*
>
> *Regarding my other comments (#1-3, #5, #7-8), I have no issues with the response provided this time.*
>
> ***Reviewer 3 Comments to the Author***
>
> *The revision substantially improves the manuscript, and most of concerns have been addressed. I recommend acceptance.*
>
> ***Reviewer 4 Comments to the Author***
>
> *The authors' changes made for statistical clarity are appreciated. Please set the decimal precision of your p-values (presently varying between 1 and 4 values) to a constant; the small sample size strongly suggests a value of 2 or 3 is appropriate. That will of course modify manuscript line 330 as well: note that the assignment of asterisk choices of 0.005 and 0.0005 is a bit nonstandard.*
>
> *Kindly supply the t values wherever t-tests are referenced in the narrative (the convention is "t(df)=x.xxx,p=0.yyy"). The same holds for Mann-Whitney's U. In line 407 and in the new final Results paragraph lines 454-460, please include the test used and its statistic as well as the p-value.*

This email was sent to tonsfelk@ohsu.edu by John Wiley & Sons, Inc.
111 River Street, Hoboken, NJ 07030 USA. 877-762-2974.
For more information, please see our privacy policy.
