# Supplementary material for: The Transcription Factors Six3 and Six6 in Neuromedin‐S Neurons Differentially Affect Circadian Rhythms
Source: J Neurosci Res. 2026 Apr 5;104(4):e70123. doi: 10.1002/jnr.70123 (PMC13051014; doi:10.1002/jnr.70123)
Supplement: Supplementary file 1 — Data S1: jnr70123‐sup‐0001‐Supinfo.zip. [file rev-3-editor-1-recommendation.pdf › rev-3-editor-1-recommendation.pdf]

WILEY

Dear Dr. Larry S. Sherman,

Dr. Tamara Franklin has recommended accepting "The transcription factors Six3 and Six6 in neuromedin-S neurons differentially affect circadian rhythms." by Karen J. Tonsfeldt.

The handling editor provided the following comments:

> “
> *Thank you for your careful revisions. All reviewer comments have now been addressed.*

To review this recommendation and submit your input, please visit the manuscript details page.

MANUSCRIPT DETAILS

Kind regards,
Journal of Neuroscience Research

This email was sent to shermanl@ohsu.edu by John Wiley & Sons, Inc.
111 River Street, Hoboken, NJ 07030 USA. 877-762-2974.
For more information, please see our privacy policy.
