# Supplementary material for: The Transcription Factors Six3 and Six6 in Neuromedin‐S Neurons Differentially Affect Circadian Rhythms
Source: J Neurosci Res. 2026 Apr 5;104(4):e70123. doi: 10.1002/jnr.70123 (PMC13051014; doi:10.1002/jnr.70123)
Supplement: Supplementary file 1 — Data S1: jnr70123‐sup‐0001‐Supinfo.zip. [file rev-3-editor-decision-letter.pdf › rev-3-editor-decision-letter.pdf]

WILEY

Dear Dr. Karen J. Tonsfeldt,

We are delighted to inform you that your manuscript jnr-2025-Aug-12634, titled "The transcription factors Six3 and Six6 in neuromedin-S neurons differentially affect circadian rhythms.", has been accepted for publication in Journal of Neuroscience Research.

You can find reviewer comments at the bottom of this email, if applicable.

What happens now?

We will now check that all of your files are complete before passing them to our production team for processing. We will let you know soon if we require any further information.

Once the manuscript has been sent to production, Wiley will email the corresponding author with information about:

- Signing the license
- Paying any applicable article publication charges (APCs)
- Reviewing manuscript proofs

To learn more, read Wiley's publication guide for authors.

If your submission contains supporting information, please read Wiley's supporting information FAQs. Supporting information is not copyedited, checked, or changed from its original format. If you notice an error, please get in touch with us as soon as possible.

Thank you for choosing to publish with Journal of Neuroscience Research.

MANUSCRIPT DETAILS

Kind regards,
Dr. Larry S. Sherman
Journal of Neuroscience Research

P.S. – You can help your research get the attention it deserves! Wiley Editing Services offers professional video abstract and infographic creation to help you promote your research. You can also read Wiley’s free Promotion Guide for best-practice recommendations on effectively promoting your work.

Handling editor comment:

> “
>
> *Thank you for your careful revisions. All reviewer comments have now been addressed.*

Reviewer comments:

> “
>
> ***Reviewer 1 Comments to the Author***
>
> > *Authors have satisfactorily addressed prior statistical concerns*
>
> ***Reviewer 2 Comments to the Author***
>
> > *The authors have adequately addressed all my concerns in this revision. I consider it appropriate to accept this paper.*

This email was sent to tonsfelk@ohsu.edu by John Wiley & Sons, Inc.
111 River Street, Hoboken, NJ 07030 USA. 877-762-2974.
For more information, please see our privacy policy.
