# Supplementary material for: The Transcription Factors Six3 and Six6 in Neuromedin‐S Neurons Differentially Affect Circadian Rhythms
Source: J Neurosci Res. 2026 Apr 5;104(4):e70123. doi: 10.1002/jnr.70123 (PMC13051014; doi:10.1002/jnr.70123)
Supplement: Supplementary file 1 — Data S1: jnr70123‐sup‐0001‐Supinfo.zip. [file JNR-104-e70123-s001.zip › rev-1-editor-decision-letter-attachment-1.docx]

**Title**

Author names

_____________________________________________________________________________________

| Review timeline: | Submission date:  Editorial Decision:  Revision Received:  Editorial Decision:  Revision Received:  Accepted: |  |
| --- | --- | --- |
|  |  |  |

Editor:

Reviewer 1:

Reviewer 2:

_____________________________________________________________________________________

1st Editorial Decision ____________________________________________________________________________________

_____________________________________________________________________________________

Authors’ Response

_____________________________________________________________________________________

2^nd^ Editorial Decision

_____________________________________________________________________________________

Authors’ Response

_____________________________________________________________________________________

3^rd^ Editorial Decision

_____________________________________________________________________________________

Authors’ Response
